# Metabolic Reprogramming of Cancer Cells and Therapeutics Targeting Cancer Metabolism

**DOI:** 10.1002/cam4.71244

**Published:** 2025-09-16

**Authors:** Jilsy M. J. Punnasseril, Abdul Auwal, Vinod Gopalan, Alfred King‐Yin Lam, Farhadul Islam

**Affiliations:** ^1^ School of Medicine & Dentistry Griffith University, Gold Coast Campus Southport Queensland Australia; ^2^ Department of Biochemistry & Molecular Biology Rajshahi University Rajshahi Bangladesh

**Keywords:** cancer metabolism, drug resistance, glucose metabolism, nucleotide metabolism, therapeutics, Warburg effect

## Abstract

**Background:**

Cancer metabolism is a field focused on the unique alterations in metabolic pathways that occur in cancer cells, distinguishing them from the metabolic processes in normal cells.

**Methods:**

An extensive review of the current literature on the metabolic adaptation of cancer cells was carried out in the current study.

**Results:**

The rapidly proliferating cells require high levels of molecules, such as glucose, amino acids, lipids, and nucleotides, along with increased energy demand (ATP). These requirements are met through alterations in the processes involving glucose, amino acid, lipid, and nucleotide metabolism. Modifications in glucose metabolism in cancer cells involve changes in glucose uptake, glycolysis, the pentose phosphate pathway, and the tricarboxylic acid cycle. Similarly, alterations in amino acid metabolism in cancer cells relate to upregulated amino acid transport and glutaminolysis. Cancer cells also have increased lipid intake from the extracellular microenvironment, upregulated lipogenesis, and enhanced lipid storage and mobilization from intracellular lipid droplets. These rapidly proliferating cells also achieve their increased demand for nucleotides by changing the expression of enzymes in the salvage and de novo nucleotide pathways. Consequently, these metabolic processes are targets for developing cancer therapeutics. However, it is important to note that the metabolic changes in cancer cells can also contribute to resistance against various cancer therapies.

**Conclusion:**

This review will explore the various ways in which cancer cells reprogram metabolic processes to sustain rapid proliferation and survival. The information presented in this report could help in the therapeutics designed to target them, and the challenges of cancer drug resistance arising from these metabolic adaptations.

**Abbreviations**
^18^FDG: 18‐fluorodeoxyglucose
27HC: 27‐hydroxycholesterol
2‐DG: 2‐deoxy‐d‐glucose
ADI‐PEG 20: pegylated arginine deiminase
ADP: adenosine diphosphate
ALL: acute lymphoblastic leukemia
BCAA: branched‐chain amino acid
BCAT1: branched‐chain amino acid transaminase 1
CAT: carnitine‐acylcarnitine translocase
dTMP: deoxythymidine monophosphate
dUMP: deoxyuridine monophosphate
EGCG: epigallocatechin‐3‐gallate
GLUD: glutamate dehydrogenase
GLUT: glucose transporter
GMP: guanosine monophosphate
hmdU: hydroxymethyl‐deoxyuridine
HMG‐CoA: β‐hydroxy β‐methylglutaryl‐CoA
HPRT: hypoxanthine guanine phosphoribosyltransferase
IDH: isocitrate dehydrogenase
IMP: inosine monophosphate
KRAS: Kirsten Rat Sarcoma
LDH: lactate dehydrogenase
LDHA: lactate dehydrogenase A
LDL: low‐density lipoprotein
NER: nucleotide excision repair
PEP: phosphoenolpyruvate
PFK: phosphofructokinase
PFKFB3: 6‐Phosphofructo‐2‐kinase/fructose‐2,6‐bisphosphatase 3
PHGDH: phosphoglycerate dehydrogenase
PRPP: phosphoribosyl pyrophosphate
PSAT: phosphoserine transaminase
RCC: renal cell carcinoma
RNA: ribonucleic acid
SLC: solute carriers
SLC7A11‐AS1: SLC7A11‐Antisense RNA 1
SREBP: Sterol regulatory element binding protein
Tap73: transcriptionally active form of the p73 protein
TCA: tricarboxylic acid
TGCT: testicular germ cell tumors
TK1: thymidine kinase 1
TKTL: transketolase‐like
TME: tumor microenvironment
TOFA: 5‐(tetradecyloxy)‐2‐furoic acid
TYMS: thymidylate synthase
XPC: Xeroderma pigmentosum complementation group C protein
YAP: Yes‐associated protein

## 1 Introduction

Cancer caused approximately 10 million fatalities in 2020, which is nearly one in every six deaths [1]. Uncontrolled cellular proliferation is the key characteristic of cancer cells. These constantly reproducing cells require significant quantities of biomolecules, including glucose, amino acids, lipids, nucleotides, and energy such as ATP. Cancer cells achieve this high demand by modifying their energy metabolism [2].

Glucose metabolism alterations in cancer cells involve glucose uptake and glycolysis. Normal cells primarily undergo oxidative phosphorylation in the presence of oxygen to yield ATP; however, increased glucose uptake and metabolism to lactate, even in the presence of adequate oxygen levels (i.e., “Warburg effect” or “aerobic glycolysis”), are widely known characteristics of cancer cells [3, 4, 5]. Low oxygen tension in the tumor microenvironment, that is, in the middle of solid tumors, is a common hallmark of cancer growth. Hypoxia is a key microenvironmental factor that influences a tumor's growth and aggressiveness. Oxygen concentration influences a cell's tendency toward specific metabolic pathways, impacting its proliferation and invasiveness. Under hypoxia, tumor cells favour glycolysis as a source of ATP. However, even in the presence of adequate oxygen, “aerobic glycolysis” quickly produces energy and metabolic intermediates that support cancer cell growth, proliferation, and the expression of adhesion molecules, despite it being less energy‐efficient than oxidative phosphorylation [6, 7]. Thus, cancer cells benefit significantly from stimulating aerobic glycolysis as opposed to oxidative phosphorylation, as seen in normal cells. This favoring of aerobic glycolysis is executed in cancer cells through the upregulation of glycolytic enzymes, which will be further discussed below.

Further pertaining to glucose metabolism, the pentose phosphate pathway (PPP) and the tricarboxylic acid (TCA) cycle are also altered in cancer cells. The PPP has two phases: the oxidative phase and the nonoxidative phase. Although the PPP is involved in glucose metabolism, these two phases are involved in aiding other metabolic pathways. For example, the oxidative phase allows for increased nucleic acid synthesis and suppression of oxidative stress, and the nonoxidative phase raises certain amino acid levels [8, 9]. In cancer cells, enzymes such as pyruvate kinase muscle isoform M2 (PKM2) and glucose‐6‐phosphate dehydrogenase (G6PD) are upregulated to facilitate the oxidative phase in preventing apoptosis of cancer cells [8, 10, 11, 12]. Transketolase enzymes involved in the nonoxidative phase are upregulated in various cancers to aid cancer cell proliferation and metastasis [13, 14, 15, 16]. Additionally, the TCA cycle occurs in the mitochondria for energy, macromolecule synthesis, and redox balance [17]. In cancer, several enzymes are upregulated or mutated in this cycle, aiding cancer cell survival [18].

Essential and nonessential amino acids are required for protein synthesis. Numerous amino acid transporters, known as solute carriers (SLCs), are expressed in the plasma membrane of normal cells. Cancer cells often increase the expression of these SLCs to enhance their uptake and export of amino acids to meet their cellular needs. In particular, glutamine serves as a nitrogen source for the production of asparagine (an essential amino acid necessary for cellular function) and hexosamine (which plays a role in a branch of glycolysis), and for the synthesis of nucleotides [19, 20]. Consequently, glutaminolysis (the conversion of glutamine into TCA cycle intermediates) is elevated in many types of cancer [21]. Ultimately, in cancers, increased amino acid transport and glutaminolysis are observed to facilitate increased protein synthesis [22]. Fatty acid oxidation serves as a significant source of energy. Fatty acids are also utilized for lipid synthesis, which is employed to form membranes and modulate signaling pathways [22]. Lipid metabolism in cancer cells involves increased fatty acid synthesis to meet the increased requirements for membrane biosynthesis as part of their support for rapid proliferation, migration, invasion, and metastasis. Cancer cells also have increased lipid intake from the extracellular milieu and enhanced lipid storage and mobilization from intracellular lipid droplets [23]. The altered expression of lipid‐metabolizing enzymes, lipid transport, and cholesterol receptors will be elaborated upon below.

Nucleotides are the building blocks for DNA and RNA. In normal cells, nucleotide synthesis occurs through the salvage and de novo nucleotide pathways. Cancer cells require more nucleotides than normal cells, which is achieved through changes in enzymes in these two pathways [24]. Cancer cells seem to favor de novo nucleotide generation; however, components of the salvage pathway have also been linked to cancer progression [24]. Due to the interconnected nature of nucleotide, glucose, fatty acid, and amino acid metabolism, disruption of one metabolic pathway, whether by nutritional deprivation or other external stress, causes cancer cells to undertake another metabolic pathway for their proliferation and survival. Consequently, blocking one of these pathways proves ineffective as a cancer treatment. Instead, the focus should shift to combination therapy, which can simultaneously target multiple metabolic pathways that are enhanced in cancer cells [25]. Some of the cancer therapeutics targeting the various altered metabolic processes have received U.S. Food and Drug Administration (FDA) approval, but most are still in development. Modifications in metabolism influence the effectiveness of certain malignancies to respond to established first‐line chemotherapy treatments, and thus, metabolic rewiring has been identified as a significant mechanism of adaptive resistance [26, 27]. This review will elaborate on the various ways in which cancer cells reprogram metabolic processes to sustain rapid proliferation and survival, examples of cancer types that exhibit these altered processes, therapeutics targeting these processes, as well as the challenges of cancer drug resistance due to these altered processes.

## 2 Glucose Metabolism in Cancer Cells

Glucose serves as the primary macronutrient utilized for energy by rapidly dividing cancer cells [7]. The following sections will explore the changes in glucose metabolism in cancer cells concerning glucose uptake, glycolysis, the PPP, and the TCA cycle.

### 2.1 Glucose Uptake

Glucose metabolism in cancer cells involves enhanced glucose uptake via overexpression of glucose transporters (*GLUT*s) [3]. *GLUT*s facilitate the transport of glucose to the cytosol through the plasma membrane. There are currently 14 isoforms of the *GLUT* genes, which can be grouped into three classes [28]. Class I, the most characterized class, is made up of *GLUT*s 1–4 and *GLUT* 14 (also known as a gene duplicate of *GLUT* 3) [15]. *GLUT*s 5, 7, 9, and 11 make up class II, whereas class III is composed of *GLUT*s 6, 8, 10, 12, and 13 [28]. *GLUT* 1 has a higher affinity for glucose than other *GLUT*s, making it crucial for tissues that rely heavily on glucose for energy [28]. *GLUT* 1 has been overexpressed in various cancers, such as breast carcinoma, colorectal carcinoma, hepatocellular carcinoma, prostate carcinoma [29], renal cell carcinoma, pancreatic carcinoma, and laryngeal carcinoma [29, 30, 31, 32, 33, 34, 35]. Moreover, *GLUT* 2 has been overexpressed in cancer cells derived from the small intestine, kidney, breast, colon, and pancreas, whereas *GLUT* 3 has been overexpressed in cancer cells derived from triple‐negative breast carcinoma, choriocarcinoma, hepatocellular carcinoma, and high‐grade glioblastoma [28, 36, 37, 38]. In general, overexpression of *GLUT* 1 and/or *GLUT* 3 is associated with poor prognosis in most cancer types studied, including colorectal carcinoma, breast carcinoma, lung adenocarcinoma, squamous cell carcinoma (SCC), ovarian carcinoma, and glioblastoma [38]. Last, Adekola et al. (2019) established a critical role for *GLUT* 4 in supporting multiple myeloma cell survival and proliferation [28].

### 2.2 Glycolysis

Glycolysis, the first stage of glucose metabolism, comprises a series of nine enzyme‐catalyzed reactions that occur in the cytosol of cells, both in an aerobic and anaerobic state. This mechanism converts one glucose molecule into two pyruvate molecules while producing a small amount of ATP and NADH [2]. Pyruvate goes into the TCA cycle under aerobic circumstances and undergoes oxidative phosphorylation, resulting in the net generation of 32 ATP molecules. In anaerobic glycolysis, pyruvate stays in the cytoplasm and converts to lactate via lactate dehydrogenase (LDH), resulting in the production of 2 ATP molecules [22, 39]. ATP generated by glycolysis provides energy and is part of the building blocks to maintain cellular integrity [22].

**FIGURE 1:**
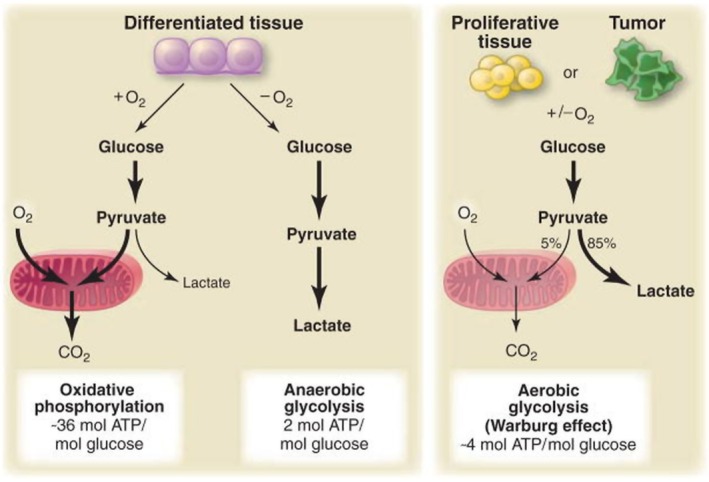
Glucose metabolism reprogramming in tumor cells. The left column demonstrates the normal glycolytic pathways. The right column denotes that in proliferating tissue or cancer cells, glucose metabolizes to lactate despite the presence of adequate oxygen (i.e., the Warburg effect). This figure is adapted from Heiden et al. with permission [40].

In the 1920s, Otto Warburg observed that compared to normal cells, cancer cells exhibited increased glucose uptake and lactic acid production, even in the presence of oxygen (i.e., under aerobic conditions) [4, 5]. This metabolic reprogramming, wherein glucose is metabolized to lactate despite the presence of sufficient oxygen, is termed the “Warburg effect” or “aerobic glycolysis” (Figure 1) [4, 5]. The Warburg effect was noted in many types of cancers as cancer types, including colorectal carcinoma, lung carcinoma, breast carcinoma, and glioma [41, 42, 43, 44, 45].

The glycolytic enzyme activity is also altered in cancer. Myc, an oncogenic transcription factor, stimulates nearly all glycolytic enzymes, such as hexokinase (HK) II, phosphofructokinase (PFK), enolase 1, pyruvate dehydrogenase kinase 1 (PDK1), and PKM2 [46]. HK, the initial enzyme in the glycolytic process, catalyzes the phosphorylation of glucose to generate glucose‐6‐phosphate (G6P) [39]. HK has been shown to have anti‐apoptotic effects; thus, overexpression of HK can mediate continued cellular proliferation [47]. Overexpression of HK has been evidenced in several cancer types, some of which include glioblastoma, medulloblastoma, breast carcinoma, stomach carcinoma, ovarian carcinoma, and cervical carcinoma [47, 48, 49, 50, 51].

PFK catalyzes the third step in glycolysis, mediating the phosphorylation of fructose‐6‐phosphate (F6P) into fructose‐1,6‐bisphosphate (F‐1,6‐BP) [4]. 6‐Phosphofructo‐2‐kinase/fructose‐2,6‐bisphosphatase 3 (PFKFB3) is an integral modulator of glycolysis, mediating the interconversion of F6P to fructose 2,6‐bisphosphate (F‐2,6‐BP) [51]. F‐2,6‐BP is a potent allosteric activator of PFK1 [4]. PFKFB3 significantly impacts multiple phases of cancer progression, including proliferation, drug resistance, and angiogenesis [52]. PFKFB3 is abnormally expressed in the brain, colon, breast, and ovarian cancer [51]. The activity of PFK, along with HK, has been shown to directly correlate with tumour aggressiveness and, thus, prognosis, such as in breast cancer [53].

The final stage of glycolysis is catalyzed by pyruvate kinase (PK), which converts phosphoenolpyruvate (PEP) and adenosine diphosphate (ADP) into pyruvate and ATP [4]. PKM2 expression is upregulated in most cancer cells. In healthy tissue, PKM2 is found in highly active tetramer and low‐active dimer forms; however, in tumor cells, it is typically found as a dimer form with limited catalytic activity. In cancer cells, this results in a change in glucose metabolism from normal oxidative phosphorylation to lactate generation, thus promoting cancer cell proliferation and growth [54]. PKM2, the isoform found in high quantities in tumor cells, is slower, resulting in PEP buildup and feedback inhibition of the glycolytic enzyme, triose phosphate isomerase. This activates the PPP since glucose‐6‐phosphate and glyceraldehyde 3‐phosphate, which are upstream glycolytic molecules, are used in the PPP [55]. The consequences of activating the PPP will be discussed below. Also, PK acts as a protein kinase and leads to cancer progression [54]. PKM2 upregulation is observed in several cancer types, including ovarian carcinomas, pancreatic carcinomas, and hepatocellular carcinoma [56, 57, 58].

Lactate dehydrogenase (LDH) is necessary for the Warburg effect. LDHA, which is activated by hypoxia or Myc, converts pyruvate to lactate in the presence of nicotinamide adenine dinucleotide + hydrogen (NADH), followed by the regeneration of NAD^+^. LDHA has been noted to be overexpressed in several cancers, such as breast carcinoma, cervical carcinoma, pancreatic carcinoma, and glioma [59, 60, 61, 62].

Pyruvate dehydrogenase kinase (PDK) is a regulatory enzyme of pyruvate dehydrogenase (PDH), which converts pyruvate into acetyl‐coenzyme A (acetyl‐CoA). PDK phosphorylates PDH, which in turn inactivates it. Malignant cells undergo metabolic changes that continuously activate PDK, which inhibits PDH, causing accumulation of pyruvate in the cytoplasm and the Warburg‐identified glycolytic alterations [63, 64]. Knockdown of PDK1 has been demonstrated to restore PDH to normal activity levels and reverse these glycolytic consequences [64]. Overexpression of PDKs, including PDK1, has been linked to the oncogenic activation of protein kinase B (Akt) and hypoxia‐inducible factor (HIF) pathways, which occur as part of the dysregulated metabolism in cancer cells [64]. Overexpression of PDK has been evidenced in various cancers, such as ovarian carcinoma, gastric carcinoma, colorectal carcinoma, and acute myeloid leukemia [65, 66, 67, 68].

### 2.3 Pentose Phosphate Pathway

The Pentose Phosphate Pathway (PPP) is a multienzyme metabolic pathway that occurs in the cytosol of cells, producing NADPH, pentoses, and ribose‐5‐phosphate [9]. It has two phases: the oxidative phase and the nonoxidative phase [9]. The oxidative phase generates several important molecules for cancer metabolism, including NADPH and ribose‐5‐phosphate [8]. The activation of the PPP through the slower activity of PKM2 (an enzyme found in high quantities in tumour cells) increases the production of NADPH [8, 55]. NADPH is an important cofactor for replacing reduced glutathione, a vital antioxidant, and thus is involved in redox homeostasis. Therefore, the increased NADPH counters the oxidative stress by ROS (reactive oxygen species) produced by the accelerated metabolism in cancer cells [55]. NADPH also promotes biosynthetic processes involving tetrahydrofolate, deoxyribonucleotides, proline, fatty acids, and cholesterol [8]. Inhibiting the processes that rely on NADPH to decrease oxidative stress may be effective in treating certain malignancies [8]. Furthermore, in the oxidative phase, G6PD catalyzes the oxidation of glucose‐6‐phosphate to 6‐phosphogluconate to produce NADPH. Tap73, a p53 family member that is frequently overexpressed in malignancies, promotes the development of G6PD, which aids in tumor growth [10, 11, 12]. The amount of G6PD frequently has a poor correlation with a patient's prognosis for cancer [69]. Interestingly, hepatocellular carcinoma cells can undergo cellular senescence due to G6PD suppression, causing intracellular oxidative stress, making cancer cells susceptible to certain chemotherapeutics, such as oxaliplatin [70, 71].

The nonoxidative phase of the PPP produces erythrose‐4‐phosphate, which can help create aromatic amino acids, including phenylalanine, tryptophan, histidine, and tyrosine [9]. In the nonoxidative phase, transaldolase and transketolase are the two important enzymes. The glycolytic intermediates, glyceraldehyde 3‐phosphate and fructose‐6‐phosphate (F6P), can be produced from ribose‐5‐phosphate [9]. Recent studies demonstrated that the transketolase family consists of the transketolase gene and two transketolase‐like (TKTL) genes, TKTL1 and TKTL2. Transketolase and TKTL1 regulate various cancer‐related events, including cancer cell proliferation, metastasis, invasion, epithelial‐mesenchymal transition, chemoradiotherapy resistance, and patient survival and prognosis [13, 14, 15, 16]. Transketolase family members are overexpressed in several malignancies, such as colorectal carcinoma, breast carcinoma, and cervical carcinoma [14, 15, 16]. As a result, transketolase could be an effective target for cancer treatment [13].

### 2.4 Tricarboxylic Acid Cycle

The TCA cycle occurs in the mitochondria and plays critical roles in cellular energy generation, macromolecule synthesis, and redox balance [17]. Various malignancies harbor mutations that compromise the integrity of this cycle, primarily affecting isocitrate dehydrogenase (IDH), succinate dehydrogenase (SDH), and fumarate hydratase (FH) [18]. The metabolic gene most commonly found to be altered in cancer is IDH1 [72]. Mutations in IDH 1 and 2 genes correlate with > 80% of low‐grade glioma cases [73] and ~20% of acute myeloid leukemia cases [72, 74]. SDH and FH act as tumor suppressors [75]. Germline SDH mutations have been noted in the neuroendocrine tumors, paragangliomas, and pheochromocytomas [75]. Germline mutations in the FH gene that deactivate the enzyme and thus alter TCA functioning can cause hereditary leiomyomatosis and renal cell carcinoma (HLRCC). HLRCC is an autosomal‐dominant condition, predisposing individuals to the development of cutaneous leiomyomas, early onset multiple uterine leiomyomas, and an aggressive form of type 2 papillary renal cell cancer [76]. In addition, studies have indicated that tumor cells can disconnect glycolysis from the TCA cycle, allowing the use of additional fuel sources, such as glutamine (an amino acid converted into TCA cycle metabolites), to meet their high metabolic demands [17, 77, 78]. The role of glutamine in cancer cells will be further discussed below.

## 3 Amino Acid Metabolism in Cancer Cells

The following sections will discuss the changes in amino acid metabolism in cancer cells, particularly amino acid transport and glutaminolysis.

### 3.1 Amino Acid Transporters

Numerous amino acid transporters, known as SLCs, are expressed in a cell's plasma membrane. SLC6A14 increases the uptake of glutamine and leucine, both of which activate mTORC1, a protein kinase involved in processes related to cell growth, proliferation, and metabolism [79, 80]. SLC6A14 upregulation was noted in cancers, including colorectal carcinoma, cervical carcinoma, breast carcinoma, and pancreatic carcinoma [81]. SLC7A11 encodes for a cystine‐glutamate antiporter, well known as system Xc‐ or xCT, which couples the uptake of cystine into cells with the efflux of glutamate from the cell. The imported cystine will be altered into two molecules of cysteine. Cysteine can participate in protein translation or be converted into methionine and participate in one‐carbon metabolism (pivotal for de novo nucleic acid synthesis, which is discussed in more detail below) and polyamine production (amines that interact with newly synthesized DNA and facilitate chromatin condensation) [82]. More critically, cysteine is a crucial substrate for glutathione production, which contributes to redox balance [83]. SLC7A11 is strongly expressed in astrocytes as well as in some other forms of cancer, such as lung, head, and neck carcinomas [83]. SLC1A5 (also known as alanine‐serine‐cysteine transporter 2 (ASCT2)) regulates glutamine uptake [81]. This glutamine transporter is crucial for cell growth and tumor formation in various malignancies, such as oral SCC, head and neck SCC, nonsmall‐cell lung carcinoma (NSCLC), breast carcinoma, and hepatocellular carcinoma [84, 85, 86]. SLC7A5 (also known as L‐type amino acid transporter 1 (LAT1)) is an antiporter that mediates the influx of neutral essential amino acids into cells in return for the outflow of intracellular essential amino acids or glutamine (a nonessential amino acid) [87]. SLC7A5 was found to be overexpressed in a range of cancers, including prostate carcinoma, lung carcinoma, and endometrial carcinoma [88, 89, 90].

### 3.2 Glutaminolysis

Glutamine can serve as a nitrogen source for the production of asparagine (an essential amino acid necessary for cellular function) and hexosamine (an amino sugar involved in a branch of glycolysis) as well as for the nitrogenous bases (purines and pyrimidines) required for nucleotide synthesis [19, 20, 22]. Glutaminolysis is a method by which cells transform glutamine into TCA cycle intermediates with the help of various enzymes. Glutamine is initially converted into glutamate by glutaminase (GLS). Glutamate is then converted into α‐ketoglutarate (α‐KG) via two distinct pathways. The first pathway occurs via the action of glutamate dehydrogenase (GLUD), whereas the second relies on transaminases, such as glutamate‐oxaloacetate transaminase (GOT), glutamate‐pyruvate transaminase (GPT), and phosphoserine transaminase (PSAT). Once generated, α‐KG is utilized for ATP production and provides carbon skeletons for the synthesis of nucleotides, fatty acids, and additional amino acids [27]. Glutaminolysis is elevated in many malignancies to provide a nitrogen source to rapidly proliferating cancer cells for amino acid synthesis [21]. For example, GLS1, the initial enzyme in glutaminolysis, is overexpressed in colorectal, esophageal, gastric, hepatocellular, and head and neck squamous cell cancer [91].

In addition to its role in amino acid metabolism, glutaminolysis is also utilized in the metabolism of other macromolecules. For example, cancer cells convert glutamine to citrate (a TCA cycle intermediate) to generate lipids and NADPH via IDH. Glutamine is also converted into malate (another TCA cycle intermediate), which is subsequently converted into pyruvate by malic enzyme, generating NADPH. This pathway contrasts with the complete oxidation of glutamine to produce ATP. Although one would expect increased complete oxidation of glutamine to ATP in cancer cells, this increased ATP production would not meet the needs of a rapidly proliferating cell as effectively. Consequently, the mitochondria shift to a greater reliance on glutamine for the TCA cycle, compensating for the reduced contribution of glucose to the TCA due to the Warburg effect. This mitochondrial reprogramming is driven by elevated levels of the oncogenic transcription factor, Myc, which enhances the expression of glutaminase and glutamine transporters, leading to increased glutaminolysis [21]. Enhancing glutamine metabolism by Myc offers the necessary components for synthesizing fatty acids and nucleotides, which are crucial for cancer cell growth and proliferation [2, 22]. Therefore, glutamine is a vital nutrient for many cancer types, particularly those driven by Myc, supporting not only amino acid metabolism but also the metabolism of other macromolecules [17].

**FIGURE 2:**
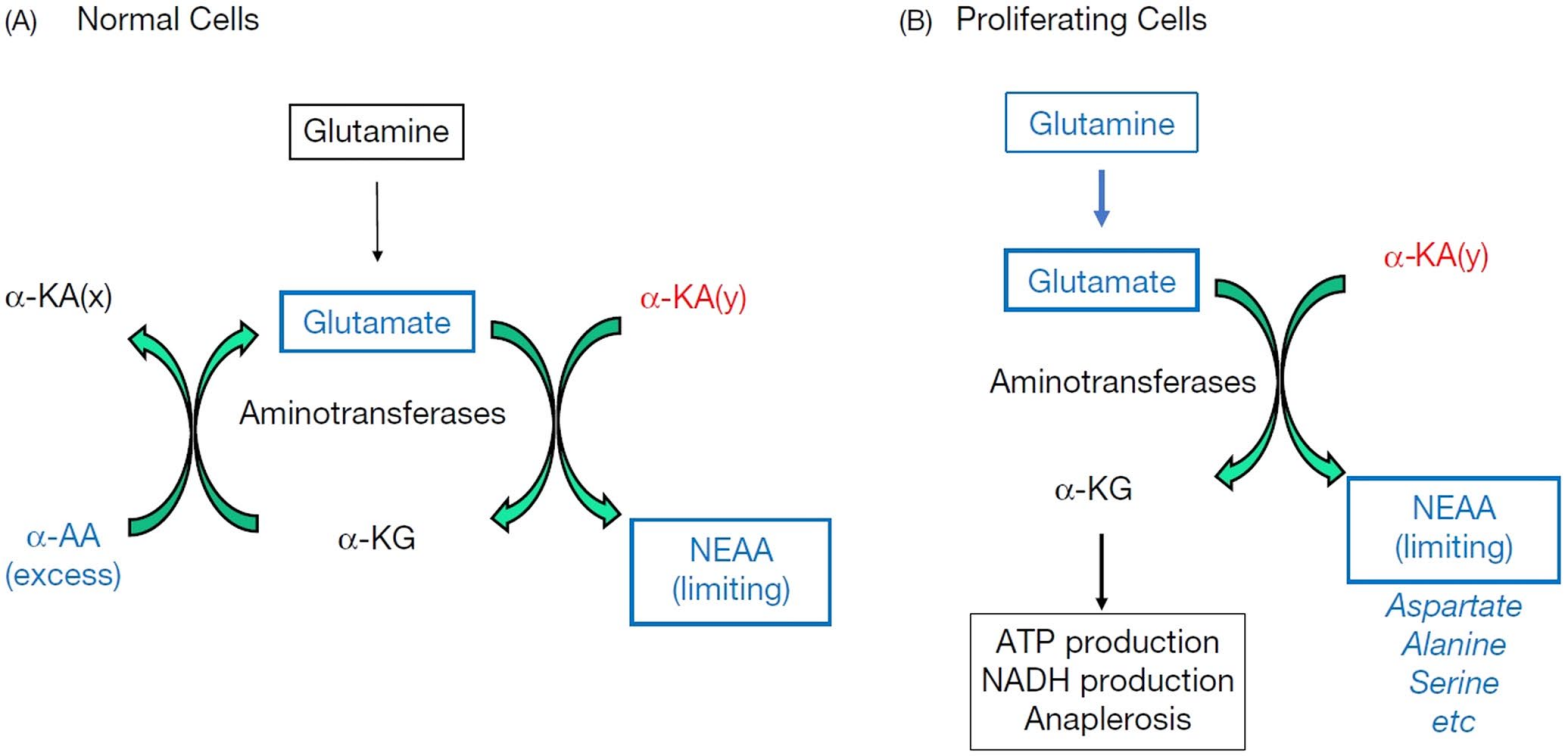
Glutamine metabolism in normal cells vs. proliferating cancer cells. (A) Glutamine metabolism in noncancerous cells. (B) In proliferating/cancer cells, glutamate is frequently used as a substrate in significant amounts through transamination to synthesize aspartate, glycine, serine, and other nonessential amino acids (NEAAs). This figure is adapted from Sang et al. with permission [83].

Moreover, as mentioned above, glutamine is initially converted into glutamate by glutaminase. In rapidly proliferating cancer cells, glutamate is typically utilized as a substrate in large quantities via transamination to synthesize aspartate, glycine, serine, and other nonessential amino acids [83] (Figure 2). Aspartate accumulation promotes cell proliferation by synthesizing nucleotides and asparagine [92, 93, 94]. Glycine is important in de novo nucleotide biosynthesis and is an important substrate for the biosynthesis of heme (a redox enzyme factor) and glutathione. Serine provides a significant supply of one‐carbon units for many processes, such as the synthesis of nucleotides, methylation of proteins, and epigenetic regulation of gene expression. Serine is also a substrate for cell membrane production [83].

## 4 Lipid Metabolism in Cancer Cells

Fatty acid oxidation serves as a significant source of energy. Additionally, fatty acids can be utilized for lipid synthesis, which is employed to form membranes and modulate signaling pathways. Fatty acid catabolism begins with converting fatty acids into acyl‐CoA to acetyl‐CoA inside mitochondria after multiple steps of conversion, and then enters the TCA to combine with oxaloacetate to form citrate. Citrate can be exported from the mitochondria and converted back to acetyl‐CoA via ATP‐citrate lyase (ACLY). Acetyl‐CoA carboxylase (ACC) uses acetyl‐CoA along with acetate to create malonyl‐CoA, which is subsequently elongated into saturated fatty acids via fatty acid synthase (FASN). Stearoyl‐CoA desaturase 1 (SCD1) is responsible for desaturating fatty acids [22]. Cancer cells exhibit irregularities in lipid metabolism, particularly in lipid transport, enzymatic activity, and cholesterol receptors compared to normal cells. Cancer cells significantly upregulate fat synthesis to meet the enhanced requirement for membrane biosynthesis, which is essential for rapid growth, migration, invasion, and metastasis. Changes in fatty acid transporters are also linked to cancer development. For example, CD36, a long‐chain free fatty acid transporter, has been found to be overexpressed in various cancers, such as oral SCC, prostate carcinoma, and colorectal carcinoma [95, 96, 97, 98, 99]. Additionally, the expression of enzymes involved in lipid metabolism is altered in cancer. Instances of overexpression have been noted for ACLY in breast carcinoma, ACC in hepatocellular carcinoma, FASN in pancreatic carcinoma, and SCD1 in ovarian cancer [100, 101, 102, 103]. Last, cancer cells necessitate large quantities of cholesterol for membrane biosynthesis because of their accelerated growth, as will be further elaborated upon in the next paragraph.

Low‐density lipoprotein (LDL) is a key lipoprotein and cholesterol carrier that facilitates cholesterol transport from the liver to peripheral tissues. Cancer cells require more cholesterol for energy than normal cells, which can lead them to enhance their cholesterol levels via receptor‐mediated endocytosis of LDL. This is particularly relevant as LDL receptors are often overexpressed in various malignancies, including hepatocellular, lung, breast, colorectal, and carcinomas [104]. Furthermore, the major metabolite of cholesterol, 27‐hydroxycholesterol (27HC), has been shown to act as a selective oestrogen receptor modulator, showing an agonistic effect in breast cancer cells, thus accelerating the formation of estrogen receptor‐positive tumors [105, 106]. Additionally, Baek et al. [107] found that consuming a cholesterol‐rich isocaloric diet alone was sufficient to enhance metastasis in numerous preclinical breast cancer models, thereby reinforcing the role of cholesterol as a contributor to metastasis. The cholesterol metabolite, 27HC, also affects myeloid cell function, leading to increased levels of polymorphonuclear neutrophils and γδ T cells and decreased cytotoxic CD8+ T cells in metastatic lesions. This alteration in immune cell composition allows cancer cells to evade immune detection at distant metastatic locations [107].

## 5 Nucleotide Metabolism in Cancer Cells

Nucleotides are the building blocks for DNA and RNA. DNA contains the instructions for the activities of the cell, while RNA translates those instructions into proteins that perform various cellular functions. Nucleotide synthesis can occur via two pathways: the salvage and de novo pathways [108]. The salvage process supports cells in recovering nucleotides and nucleobases from the intra‐ and extracellular environment through a reaction with phosphoribosyl pyrophosphate (PRPP) [24, 108]. The de novo pathway synthesizes purine and pyrimidine nucleotides by fusing carbon dioxide, amino acids, derivatives of folate, TCA cycle intermediates, ribose sugars, and PRPP [22, 108]. Cancer cells require more nucleotides than other types of cells [24]. In addition to altered glucose and amino acid metabolism, this is achieved via changes in enzymes in the salvage and de novo nucleotide pathways.

**FIGURE 3:**
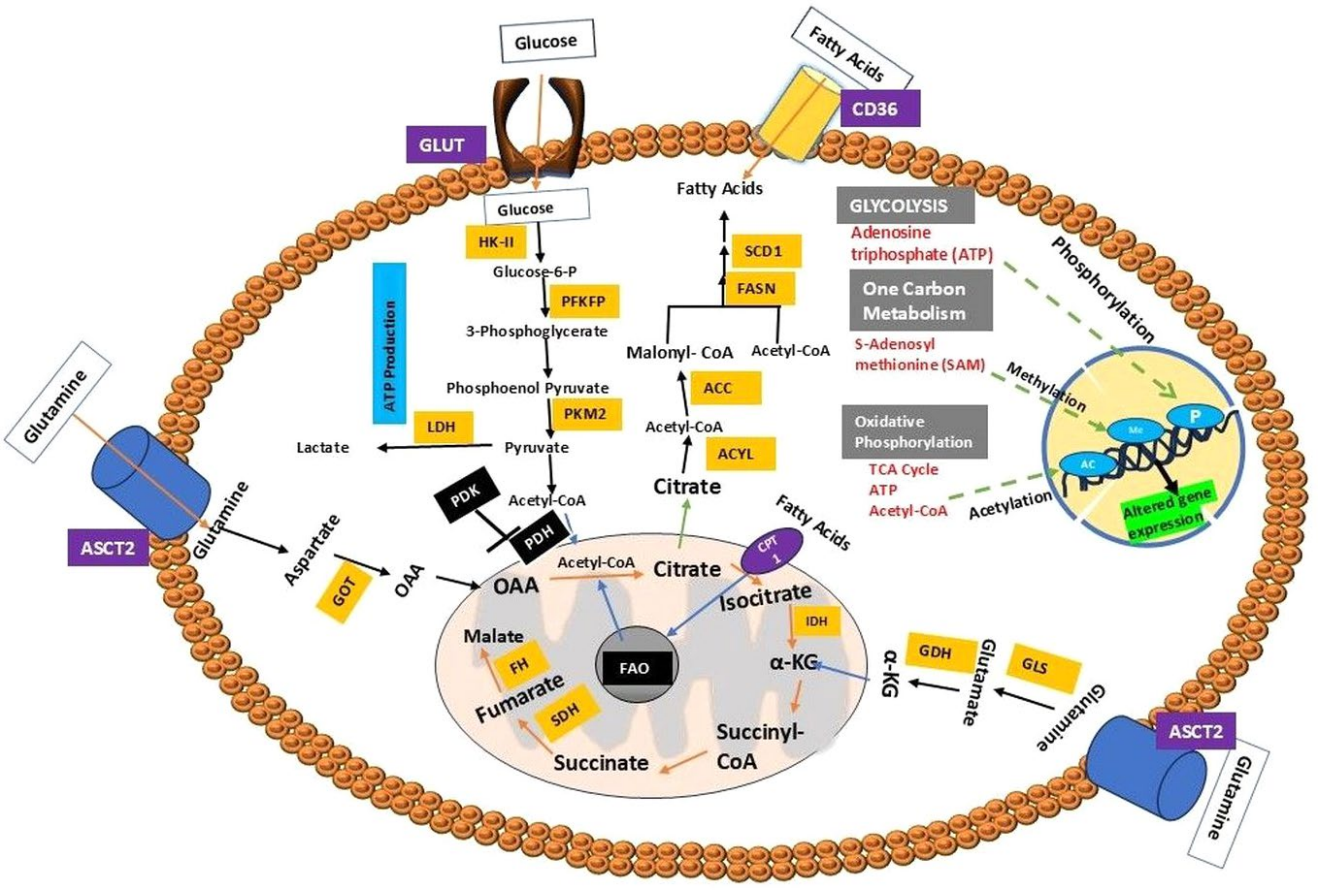
Alteration in the metabolism of cancer cells. The metabolic adaptation of a cancer cell allows it to recruit increased biosynthetic intermediates and energy required for its growth, development, and proliferation. Cancer cells have increased expression of GLUTs, allowing for greater glucose absorption. Overexpression of CD36 accelerates fatty acid import. Acetyl‐CoA is produced by β‐oxidation of fatty acids in mitochondria and converted to citrate by the enzyme citrate synthase. CPT1 facilitates the transport of fatty acids to mitochondria. Changes in lipid metabolism supply the energy essential for cancer cell growth as well as metabolites for biosynthetic pathways. In amino acid metabolism, cancer cells overexpress the glutamine transporter ASCT2. GLS hydrolyzes glutamine into glutamate. Glutamate can be converted into intermediates of the TCA cycle, including αKG. Glutamine can be transformed to aspartate, which can then be turned to OAA by the enzyme GOT, thus providing TCA cycle intermediates. In cancer, this manifests as increased glucose flux through glycolysis, substrates for one‐carbon metabolism, and increased acetyl‐CoA transport out of the mitochondria in the form of citrate, which allows for abnormal epigenetic modifications on both chromatin and DNA, potentially promoting cancer cell development.

Cancer cells seem to favor de novo nucleotide generation; however, salvage pathway components such as hypoxanthine guanine phosphoribosyltransferase (HPRT) have been linked to cancer progression [24]. It changes cellular precursors into inosine and guanine (one of the four nucleotide bases) by creating guanosine monophosphate (GMP) and inosine monophosphate (IMP) [24]. HPRT was overexpressed in lung, colon, prostate, and breast cancer tissue samples [109]. In addition, thymidine kinase 1 (TK1) mediates the alteration of thymidine to deoxythymidine monophosphate (dTMP) in the salvage pathway, whereas thymidylate synthase (TYMS) in the de novo pathway [24, 110, 111]. Interestingly, TK1 and TYMS have been acting as tumor markers for several malignancies, such as leukemia, colorectal carcinoma, lung carcinoma, breast carcinoma, and prostate carcinoma [24]. In summary, Figure 3 illustrates the metabolic reprogramming of glucose, amino acid, lipid, and nucleotide pathways as previously discussed.

## 6 Drug Resistance and Metabolic Pathways of Cancer Cells

Alterations in metabolism allow cancer cells in several malignancies to escape the response to first‐line chemotherapy treatments that have been proven effective. Thus, metabolic rewiring has been recognized as a significant mechanism of adaptive resistance [26]. This section explores examples of cancers where metabolic changes confer resistance to cancer therapies, primarily focusing on chemotherapy resistance.

### 6.1 Glucose Metabolism and Drug Resistance

Glucose is the main macronutrient that rapidly dividing cancer cells use for energy. For fast glucose production, the “Warburg effect” or “aerobic glycolysis” is observed in cancer cells. The phenomenon occurs through overexpression of glycolytic enzymes, which can lead to resistance against chemotherapy drugs. This section will explore the previously discussed enzymes involved in glucose metabolism that contribute to this resistance to certain chemotherapeutics.

One such enzyme is HK2, whose overexpression results in increased glycolytic flux—a characteristic feature of cancer cells. In hepatocellular carcinoma, heightened HK2 activity yielded an eightfold increase in resistance to cisplatin [51]. Moreover, proviral insertion in murine lymphomas 2 is a proto‐oncogene that mediates PFKFB3 phosphorylation, which can confer paclitaxel resistance in breast cancer [112]. When PFKFB3 was silenced in paclitaxel‐resistant breast cancer cells, enhanced susceptibility of these cancer cells to paclitaxel was observed [113]. PKM2 expression is also elevated in various cancer types [114]. Metformin, a medication used primarily for diabetes, inhibits PKM2, which can induce tumor cell death and enhance sensitivity to chemotherapy [115]. For example, metformin has been shown to increase the susceptibility of osteosarcoma stem cells to cisplatin by reducing PKM2 expression levels [116].

LDH catalyzes the reversible alteration of pyruvate into lactate [117]. LDHA has been shown to improve survival in tamoxifen‐resistant breast carcinoma cells. Consequently, inhibiting LDHA by pharmacological and genetic means sensitizes these breast carcinoma cells to tamoxifen [118].

G6PD, an enzyme in the PPP, was shown to be overexpressed in cisplatin‐resistant ovarian cancer cells. A study by Catanzaro et al. [119] demonstrated that when cisplatin and 6‐aminonicotinamide (a competitive G6PD inhibitor) were used together, cisplatin‐resistant cells were more susceptible to the cisplatin chemotherapy drug. Enhanced G6PD expression was also evidenced in the cisplatin‐resistant clear cell subtype of renal cell carcinoma (ccRCC) cells. ccRCC cells pretreated with 6‐aminonicotinamide helped sensitize the cancer cells to cisplatin, compared to ccRCC cells only treated with cisplatin monotherapy [120].

Chemotherapy resistance can result from the overexpression of enzymes involved in glucose metabolism. Targeting these enzymes is a strategy to combat chemotherapy resistance to improve treatment efficacy.

### 6.2 Amino Acid Metabolism and Drug Resistance

Modified amino acid metabolism offers cancer cells adaptive features to resist anticancer therapy. This resistance arises from changes in the oxidative stress levels within cancer cells, alterations in protein synthesis, modifications in the transport of amino acids, and adjustments to the enzymes involved in amino acid metabolism.

As previously stated, glutaminolysis yields glutamate, which is then used to synthesize glycine, a crucial substrate for glutathione (an important antioxidant). Cancer cells leverage antioxidants derived from glutathione to tackle the oxidative stress induced by treatments, leading to resistance against drugs [121]. For example, in HCC cells that are resistant to the TKI, sorafenib, an increase in glutamine metabolism aids in the survival of these resistant cancer cells through the NADPH‐dependent glutathione redox system. Inhibiting this metabolic pathway sensitizes sorafenib‐resistant hepatocellular carcinoma cells to sorafenib [122]. Another example is seen in cisplatin‐resistant NSCLC cells. Glutamine helps mitigate the oxidative stress caused by cisplatin through the production of glutathione, and cells that are resistant to cisplatin show increased sensitivity to the lack of glutamine [123].

Bortezomib was the first proteasome inhibitor introduced for clinical use. It plays a crucial role in the treatment of multiple myeloma. Proteasome inhibitors lead to an overproduction of ROS, which disrupts the balance between protein synthesis and degradation, ultimately resulting in the apoptosis of cancer cells. Bortezomib resistance is linked to mutations in the proteasomal bortezomib‐binding pocket as well as an overexpression of the proteasomal machinery, both of which reduce the drug's effectiveness [26].

xCT, the antiporter that couples the uptake of cystine into cells with the efflux of glutamate from the cell, was shown to be upregulated in cisplatin‐resistant gastric carcinoma cells. Upregulation of SLC7A11‐Antisense RNA 1 (SLC7A11‐AS1) blocks xCT, aiding in reversing cisplatin chemoresistance both in vitro and in vivo. This effectively weakens the growth of gastric carcinoma cells, reduces intracellular glutathione biosynthesis, and enhances intracellular ROS [124].

Argininosuccinate synthetase (ASS1) is an enzyme that catalyzes the production of argininosuccinate from citrulline and aspartate. Loss of ASS1 causes aspartate buildup, which promotes cell proliferation via nucleotide and asparagine generation [22]. ASS1 is significantly downregulated in cisplatin‐resistant bladder carcinoma cells. Decitabine reestablished cisplatin susceptibility in resistant bladder carcinoma cells by increasing ASS1 expression. The addition of exogenous arginine deiminase, administered as ADI‐PEG 20 (pegylated arginine deiminase), also increased ASS1 expression and accelerated cisplatin‐induced apoptosis [114].

Moreover, Thewes et al. demonstrated that compared to their antiestrogen‐sensitive breast carcinoma cell line, the antiestrogen‐resistant breast carcinoma cells had upregulated branched‐chain amino acid transaminase 1 (BCAT1). BCAT1 catalyzes the initial step in the degradation of branched‐chain amino acids. When BCAT1 was turned off in an orthotopic triple‐negative xenograft model, the tumor volume dropped by a large amount in vivo [125]. The alterations in amino acid metabolism in cancer can be a promising target for anticancer medications.

### 6.3 Lipid Metabolism and Drug Resistance

Altered lipid metabolism seen in treatment‐resistant cancer cells significantly contributes to their survival and leads to unfavorable outcomes. This section provides examples of how targeting specific alterations in lipid metabolism confers sensitivity to certain cancer therapeutics.

Sterol regulatory element binding proteins (SREBPs) are transcription factors that control lipid homeostasis. SREBP1 encourages the synthesis of fatty acids and triglycerides, causing an accumulation of lipid droplets inside the cells. SREBP1 was found to be overexpressed in gemcitabine‐resistant colorectal carcinoma samples, and SREBP1 overexpression was linked to lower patient survival [126]. Interestingly, inhibiting SREBP1 enhanced chemosensitivity to gemcitabine in colorectal carcinoma cells [126].

Cancer stem‐like cells of prostate origin have been found to exhibit the high activity of HMG‐CoA reductase, which is a rate‐limiting enzyme in cholesterol synthesis. This increased activity promotes resistance against docetaxel through increased expression of the oncogene, Yes‐associated protein (YAP). Research has demonstrated that the combined use of valproic acid and simvastatin (an HMG‐CoA reductase inhibitor) can sensitize prostate carcinoma to docetaxel by targeting cancer stem cells through YAP inhibition [127].

Sorafenib is a kinase inhibitor that treats advanced hepatocellular carcinoma. However, sorafenib resistance usually develops after a few months of treatment. Sheung et al. (2022) demonstrated that sorafenib‐resistant cells expressed high levels of FASN. Inhibiting FASN with orlistat allowed for sensitization of the cancer cells to sorafenib [128].

### 6.4 Nucleotide Metabolism and Drug Resistance

As previously mentioned, nucleotides are the building blocks for DNA and RNA. Numerous therapeutics target the mechanisms that preserve DNA integrity in cancer cells, which have developed resistance to these therapies. This section will explore examples of resistance mechanisms pertaining to nucleotide, DNA, and RNA metabolism.

Mutations in the *BRCA1* or *BRCA2* tumor suppressor genes increase the risk of breast carcinoma and ovarian carcinoma. Cells with BRCA defects show significant chromosomal rearrangements due to their impaired DNA repair capabilities. In clinical practice, these malignancies can be treated using inhibitors that target poly[ADP‐ribose] polymerase (PARP), an enzyme responsible for repairing DNA in damaged cells. However, although there may be an initial positive response, many tumors eventually develop resistance to PARP inhibitors, resulting in more aggressive tumor growth. Research has indicated that inhibiting 2′‐Deoxynucleoside 5′‐Phosphate N‐Hydrolase 1 (DNPH1), a protein that eliminates the cytotoxic nucleotide hydroxymethyl‐deoxyuridine (hmdU) monophosphate, can enhance the sensitivity of *BRCA*‐mutated breast carcinoma cells to PARP inhibitors [129].

Cisplatin's efficacy depends on its binding to DNA and the generation of cytotoxic DNA damage to eradicate cancer cells. Nucleotide excision repair (NER), a DNA repair system, is a significant resistance mechanism against cisplatin in tumor cells. NER proteins can detect DNA damage caused by cisplatin and repair it, which neutralizes cisplatin's cytotoxicity, thereby contributing to the development of drug resistance. Studies in testicular germ cell tumors (TGCT) have shown minimal NER activity in TGCT cell extracts. The intrinsic NER abnormalities in TGCT cells are associated with increased cisplatin sensitivity and a high cure rate among TGCT patients. This concept of low NER sensitising cancer cells to cisplatin was used to discover that inhibiting the production of XPC protein (an NER protein) by small interfering RNA (siRNA) knockdown enhances cisplatin sensitivity [130].

Methotrexate inhibits cell division by inhibiting folate‐related enzymes, which are important in pyrimidine and purine nucleotide biosynthesis [131]. Thus, methotrexate is a chemotherapy drug used to treat various malignancies. However, MDA‐MB‐231, a breast carcinoma cell line, has evolved resistance to it due to a shortage of the methotrexate transport protein and diminished folate carrier. Raut et al. [132] developed a pH‐responsive zinc oxide nanocarrier, which aids in the efficient delivery of methotrexate to MDA‐MB‐231 breast cancer cells, thereby overcoming the methotrexate resistance.

Ultimately, cancer cells' ability to reorganize the metabolic pathways may hinder precision therapies by promoting resistance. Therefore, combination medications, which inhibit multiple pathways, may be more beneficial than single‐agent therapy [22]. Therefore, a better understanding of the metabolic rewiring processes in cancer cells will aid in developing anticancer therapeutics.

## 7 Cancer Therapies Targeting Altered Metabolic Processes

Several metabolic pathways are altered in tumor cells to manage the requirement for biomolecules and energy to sustain rapid cell proliferation in tumor growth and progression. This section explores the cancer therapies targeting these altered metabolic processes.

### 7.1 Cancer Therapeutics Targeting Glucose Metabolism

The most prevalent metabolic adaptation in cancer cells is altered glucose metabolism. Thus, inhibiting altered glucose metabolism by targeting GLUTs, glucose metabolizing enzymes, can halt the spread of cancer. Glucose‐metabolizing enzymes used as therapeutic targets could present novel perspectives to consider for cancer treatment. Many glucose‐metabolizing enzyme inhibitors have been and are still being explored as anticancer drugs [133]. Table 1 describes various cancer therapeutics targeting glucose metabolism.

**TABLE 1:** Cancer therapeutics targeting glucose metabolism.

Agent	Metabolic target	Indications	Concentration/dose of agent	Study model	Outcomes	References
WZB115, WZB117	GLUT 1 inhibitor	Breast carcinoma, lung carcinoma	5 and 10 μmol/L; 10 μM	In vitro	Increased carcinoma death	[134, 135]
STF‐31	GLUT 1 inhibitor	RCC	11.6 mg/kg	In vitro and in vivo	Glucose uptake inhibition	[33]
Fasentin	GLUT 1 inhibitor	Prostate carcinoma, lymphoma	15,20,30,40,60,80 μM	In vitro	Glucose uptake inhibition	[136]
Phloretin	GLUT 1 or 2½ inhibitor	HCC, breast, prostate, lung, colorectal cancers	10–150 μM 25 mg/kg	Both in vivo and in vitro	Inhibited tumor growth and metastasis	[137]
Ritonavir	GLUT 4 inhibitor	Multiple myeloma, breast carcinoma, CLL	5–100 μM	In vitro	Induction of significant cell death	[138]
2‐deoxy‐D‐glucose (2DG)	HK inhibitor	Prostate carcinoma	8 mM	In vitro	Inhibition of glucose metabolism and induction of cell death	[139]
Glloflavin	LDHA inhibitor	Breast carcinoma	0–250 μM	In vitro	Reduction of cancer cell proliferation by inhibiting glycolysis and ATP generation	[140]
Polyphenon E	LDHA inhibitor	Breast carcinoma, colon carcinoma	0‐50 μg/mL	In vitro	Inhibition of cancer cell growth	[141]
6‐aminonico‐tinamide (6‐AN)	G6PD inhibitor	RCC, lung cancer	1–1000 μM	In vitro	Inhibition of glucose consumption	[142]
Shikonin	PKM2 inhibitor	Breast carcinoma, skin carcinoma, bladder carcinoma	0.5, 1.00, and 1.50 μM	In vitro	Significant inhibition of growth and migration	[143]
Orlistat	PKM2 inhibitor	Ovarian carcinoma	20 mM	In vitro	Significantly downregulated PKM2 enzyme and cancer cells	[144]
3‐(3‐pyridinyl)‐1‐(4‐pyridinyl)‐2‐propen‐1‐one (3PO)	PFKFB3 inhibitor	Lung carcinoma, pancreatic carcinoma, melanoma	5–200 μM	In vitro	Inhibition of glucose uptake and cell division	[145]
PFK15	PFKFB3 inhibitor	RCC, HCC, CRC, gastric carcinoma	0–20 μM; 25 mg/kg	Both in vivo and in vitro	Significantly inhibited invasion and reduced tumor weight and volume	[146]
PFK158	PFKFB3 inhibitor	Lung carcinoma, ovarian carcinoma	0–15 μM; 25 mg/kg	Both in vivo and in vitro	Decreased glucose uptake and apoptosis induction	[147]
Ivosidenib	Mutant IDH1 inhibitor	AML	100–1200 mg daily	FDA‐approved	Inhibited mutant IDH1 chondrosarcomas	[148]
Enasidenib	Mutant IDH2 inhibitor	AML	50–650 mg/day	FDA‐approved	Improved induced hematologic responses	[149]

Abbreviations: AML, acute myeloid leukemia; CLL, chronic lymphocytic leukemia; CRC, colorectal carcinoma; G6PD, glucose‐6‐phosphate dehydrogenase; GLUT, glucose transporter; HCC, hepatocellular carcinoma; HK, hexokinase; IDH, isocitrate dehydrogenase; LDHA, lactate dehydrogenase; PFK, phosphofructokinase; PFKFB3, 6‐Phosphofructo‐2‐kinase/fructose‐2,6‐bisphosphatase 3; PKM2, pyruvate kinase muscle isozyme 2; RCC, renal cell carcinoma.

*GLUT* inhibitors are a promising therapy that has been researched in many investigations. For instance, a small‐molecule inhibitor of *GLUT* 1, namely STF‐31, demonstrated activity against renal cell carcinoma xenografts in vivo and showed the hallmarks of a *GLUT* inhibitor. Nevertheless, this agent has off‐target consequences. In particular, nicotinamide phosphoribosyltransferase (NAMPT) is also inhibited by STF‐31. The reduction of tumor growth by STF‐31 was shown by expressing a drug‐resistant NAMPT mutant or by adding nicotinic acid, suggesting that STF‐31 reduces tumor growth through mechanisms other than *GLUT* 1 inhibition [22]. Moreover, WZB117 is a bis‐hydroxybenzoate that suppresses cancer cell growth by preventing glucose transport via *GLUT* 1's glucose binding site [133]. WZB117 also has the potential to work in synergy with other anti‐cancer drugs, such as cisplatin or paclitaxel, to enhance their effects on breast and lung cancer cells [150]. Some natural chemicals, such as genistein, fasentin, apigenin, and phloretin, also demonstrated *GLUT* suppression and hence inhibited the proliferation of cancer cells. Phloretin, a polyphenol, has been shown to inhibit *GLUT* 2 in triple‐negative breast cancer, suppressing tumor development and metastasis [133]. In addition, phloretin can block *GLUT* 1, which is excessively expressed in the hypoxic regions of resistant colon cancer cells. This activates p53‐mediated signaling pathways, triggering apoptosis and ultimately hindering the growth of these resistant cancer cells [151]. Fasentin has been demonstrated to reduce resistance to caspase activation and block glucose uptake, both of which influence the chemoresistance of cancer cells. Furthermore, fasentin uses a metabolism that is not dependent on glucose to prevent angiogenesis [152].

The initial step of glycolysis is catalyzed by HK. As it is both independently and cooperatively activated by HIF1 and Myc, HK2 expression is increased in cancer [153]. Since HK2 loss reduces carcinogenesis in vivo, it is a potential subject for treatment. 2‐deoxy‐d‐glucose (2‐DG) is an HK2 antagonist, thereby inhibiting glycolysis [154]. Preclinical research has shown that 2‐DG strongly suppresses ATP synthesis and glycolysis [154]. Since its reintroduction, 2‐DG has been used in combination therapies, where it works in concert with other anticancer drugs to provide synergistic anticancer effects [133]. 2‐DG has been utilized in several clinical trials as an adjuvant to clinical chemotherapeutic drugs for a variety of malignancies, including gliomas, lung carcinoma, ovarian carcinoma, prostate carcinoma, and breast carcinoma [155, 156, 157].

The final stage of glucose metabolism is catalyzed by LDH, which converts pyruvate to lactate in a reversible manner. The pH in the tumor microenvironment (TME) is impacted by lactate buildup. Lactate is a pro‐inflammatory and immunosuppressive mediator that accelerates the development of malignant tumors. It has been indicated that elevated lactate levels are linked to early distant cancer metastasis [158]. Thus, there is research being conducted to investigate LDH inhibitors. Galloflavin has been shown to connect with free LDHA and block glycolysis in breast cancer cells, thereby reducing cancer development [140]. Oxamate, a competitive LDHA inhibitor, has been shown to suppress the conversion of pyruvate to lactate, thus decreasing proliferation of prostate and T‐cell acute lymphoblastic leukemia [133]. Notably, oxidative cancer cells (cancers that rely on mitochondrial oxidative phosphorylation) are less vulnerable to LDHA inhibitors. Certain glycolytic cancer cells (cancers that rely on upregulated glycolysis) can enhance oxidative phosphorylation while suppressing glycolysis, resulting in resistance to LDHA inhibitors. Thus, LDHA inhibitors can be combined with oxidative phosphorylation inhibitors, such as phenylephrine, to produce an anticancer action [159]. Shikonin, an active component derived from the comfrey plant, is a powerful and specific PKM2 inhibitor, and Shikonin's analog, alkannin, showed cancer‐inhibiting activity via PKM2 inhibition. Shikonin inhibits PKM2 activity in advanced bladder cancer cells and colon cancer, reducing platinum resistance. In addition, it reverses cisplatin resistance dose dependently in cervical cancer cells [160].

PFK catalyzes the third step in glycolysis, which converts F6P to F‐1,6‐BP. F‐2,6‐BP is a potent allosteric activator of PFK1 [4]. Elevated F‐2,6‐BP levels can activate PFK, allowing cancer cells to proliferate. PFKFB is an enzyme that functions as both a kinase and a phosphatase, and the amount of F‐2,6‐BP is determined by the relative activity of the two enzymes. Consequently, lowering F‐2,6‐BP levels inhibits the kinase activity of PFKFB while maintaining the phosphatase activity, which can reduce PFK1 function and prevent the formation of cancer [133]. Targeted therapy for many cancers is based on PFKFB3, which is underexpressed in normal tissues and overexpressed in malignancies of the breast, colon, ovaries, and thyroid [161]. PFK15 has PFKFB3 inhibitory activity and has been shown to have considerable anticancer efficacy in xenografted tumors, decreasing uptake of 18‐fluorodeoxyglucose (^18^FDG) (a marker for glucose uptake by tissue) and F‐2,6‐BP levels. Furthermore, PFK15 promotes apoptosis in transformed cancer cells, both in vivo and in vitro [162]. Several investigations have shown that PFK15 and PFK158 can interact with targeted and chemotherapeutic drugs [133, 162]. The combination of CTLA‐4 antibody and PFK158 can effectively reduce cancer development, indicating a promising future for immunotherapy combined with targeted glucose metabolic therapy [133].

IDH is a family of metabolic enzymes that play key roles in the TCA cycle. Specifically, they catalyze the oxidative decarboxylation of isocitrate to produce α‐KG and reduce NAD^+^ and NADP^+^ to NADH and NADPH, respectively. IDH1/2 mutations are known to increase the development of several malignancies, including lymphoma and glioma. The FDA approved the use of Ivosidenib, which targets MUTANT IDH1, and enasidenib, which targets IDH2 for acute myeloid leukemia [161, 162, 163, 164, 165].

### 7.2 Cancer Therapeutics Targeting Amino Acid Metabolism

To meet their energy needs, tumor cells require significant amounts of amino acids throughout their proliferation. Antimetabolites were employed in the early phases of cancer treatment to inhibit tumor metabolism. The growth, development, and proliferation of cancer cells can be influenced by decreasing blood amino acid levels, restricting amino acid transport, or catabolizing amino acid‐metabolizing enzymes. A number of therapeutics targeting the amino acid metabolism of cancer cells are being developed to manage patients with various cancers (Table 2).

**TABLE 2:** Cancer therapeutics targeting amino acid metabolism.

Agent	Metabolic target	Indication	Concentration/dose of agent	Study model	Outcomes	References
CB‐839	Glutaminase inhibitor	TNBC, leukemia, CRC, RCC	1 μM and 200 mg/kg	Both in vivo and in vitro	Potentially improve immune function in TME and killing tumor cells	[166, 167]
968	Glutaminase inhibitor	Breast carcinoma	0–50 μM	In vitro	Promising inhibited cell proliferation	[168, 169]
IPN60090	Glutaminase inhibitor	Advanced solid tumors	100 mg/kg	Clinical trials	Inhibited tumor development	[170]
DRP‐104	Glutamine‐utilizing enzymes	NSCLC, neck and head SCC and other advanced solid tumors	1–4 mg/kg	In vivo	Inhibited glutamine‐dependent nucleotide synthesis. Promotes antitumor T‐cell responses.	[171]
JPH203 (also known as KYT‐0353)	Competitive LAT1 inhibitor	Prostate carcinoma Biliary tract cancer	0.3–10 μM	In vivo	Inhibits LAT1 function through preincubation of cells with JPH203	[172, 173, 174]
Sulfasalazine	SLC7A11 inhibitor	Breast carcinoma	1 mM; 250 mg/kg	Both in vivo and in vitro	Inhibits KRAS‐mutant cancer cell inhibition	[175, 176]
V‐9302	ASCT2 inhibitor	Osteo‐sarcoma xenografts	25 μM	In vitro	Attenuates growth, proliferation of cancer cells, and apoptosis induction.	[177, 178]
NCT‐503	PHGDH inhibitor	PHGDH‐dependent breast carcinoma xenografts	10 μM	In vitro	Significant reduction in all cell models, independent of PHGDH expression level.	[179, 180]
R162	GLUD1 inhibitor	Lung carcinoma xenografts	20 μM and 20 mg/kg/day	Both in vivo and in vitro	Limits tumor progression, metastasis, and reverses drug resistance	[181, 182]
ADI‐PEG 20	Arginase	Metastatic melanoma	36 mg/m^2^	Clinical trials	Prevent metastasis	[183, 184]
Recombinant Methioninase (r‐METase)	Methionine production inhibitor	Prostate cancer Pancreatic carcinoma and melanoma xenograft nude‐mouse models	250 units	Clinical trials	Improve quality of life	[185, 186]

Abbreviations: ADI‐PEG 20, pegylated arginine deiminase; ALL, acute lymphoblastic leukemia; ASCT2, alanine‐serine‐cysteine transporter 2; CRC, colorectal cancer; GLUD, glutamate dehydrogenase; LAT1, L‐type amino acid transporter 1; NCT‐503, N‐(4,6‐Dimethylpyridin‐2‐yl)‐4‐(4‐(trifluoromethyl)benzyl)piperazine‐1‐carbothioamide; NSCLC, nonsmall‐cell lung cancer; PHGDH, phosphoglycerate dehydrogenase; PSA, prostate‐specific antigen; RCC, renal cell carcinoma; r‐METase, recombinant methioninase; SCC, squamous cell carcinoma; SLC7A11, solute carrier 7A11; TNBC, triple‐negative breast cancer.

Pharmacological GLS inhibitors have directly targeted the apoptotic pathway, which acts as a vital role in tumor cell survival. CB‐839, a GLS inhibitor, enhances the tumoricidal activity of natural killer cells by blocking glutamine used by tumor cells, boosting glutamine availability in the TME, and activating the mTOR and c‐Myc signaling pathways [187]. V‐9302, an ASCT2 inhibitor, was developed using the glutamyl anilide scaffold, which mimics glutamine. It exhibits antitumor action in vivo [22]. It has also been shown that it has in vivo effectiveness in the immunocompetent E0771 mouse model of breast cancer by increasing T cell activation, most likely due to decreased ‘glutamine steal’ by tumor cells [177]. Furthermore, V‐9302 paired with the glutaminase inhibitor, CB‐839, inhibited the growth of human liver tumor transplants [188]. Additionally, the LAT1 inhibitor, JPH203 (also known as KYT‐0353), inhibits the influx of numerous neutral amino acids. It is presently in phase I, intending to move to phase II clinical trials [189]. JPH203 has shown effectiveness in vivo against the HT‐29 colon carcinoma xenografts [22]. IPN60090, a molecule developed from the co‐crystal structure of the GLS inhibitor, BPTES, originates from a different scaffold than CB‐839. It exhibits a better pharmacokinetic profile and enhanced in vivo activity at lower doses in a patient‐derived xenograft model for NSCLC when used in conjunction with an mTORC1/2 inhibitor [170]. An increased need for methionine for tumor development, known as methionine dependency, appears to be a widespread metabolic variation in cancer. Kawaguchi et al. [185] reported that in patient‐derived xenograft nude mouse models with pancreatic cancer or melanoma, recombinant methioninase‐treated tumors exhibited a lower methionine content and were smaller in size when compared to those of the untreated group.

The plasma membrane glutamine transporter, SLC7A11, is inhibited by sulfasalazine. More potent compounds based on the scaffold of sulfasalazine have been developed and have been shown to significantly inhibit tumor growth as well as cystine and glutathione synthesis [140].

Another important area of research for potential therapeutic targets is the metabolism of branched‐chain amino acids (BCAA) One helpful prognostic sign for cancer is BCAT1, an enzyme that catalyzes the initial step in the degradation of branched‐chain amino acid metabolism [125]. Eupalinolide B is a naturally derived small molecule that has been shown to inhibit BCAT1 in triple‐negative breast cancer mouse models [190].

Phosphoglycerate dehydrogenase (PHGDH) is an enzyme that limits the rate of serine production from glucose. The small molecule inhibitor, NCT‐503, could potentially inhibit PHGDH, which is useful for studying serine production via PHGDH in various cancer types. For instance, Arlt et al. (2021) discovered an off‐target effect of NCT‐503 using a pulsed stable isotope‐resolved metabolomics method with 13C‐glucose in neuroblastoma cells and genetically PHGDH knockout clones. NCT‐503 significantly decreased glucose‐derived citrate production in neuroblastoma cells, consistent with higher integration of glucose‐derived carbons into the TCA cycle from pyruvate to malate [179].

Last, L‐asparaginase enzyme has long been used to treat children with acute lymphoblastic leukemia. Asparagine is an essential amino acid necessary for cellular function. Normal cells produce it using the enzyme L‐asparagine synthetase, which converts aspartic acid and glutamine into asparagine and glutamate. In contrast, leukemic cells exhibit low levels of this enzyme and rely on external serum asparagine for their growth. L‐asparaginase, an enzyme not found in humans, interferes with this process by breaking down serum asparagine into aspartic acid and ammonia. When there is insufficient asparagine, protein synthesis in tumor cells is hindered, ultimately leading to cell death [19].

### 7.3 Cancer Therapeutics Targeting Lipid Metabolism

Lipid metabolic reprogramming is required for tumor survival and proliferation. Therapeutic interventions that target lipid metabolic reprogramming in cancer may be an effective method of reducing tumor growth. Anticancer therapies could target lipid metabolic pathways, enzymes, and molecular processes (Table 3).

**TABLE 3:** Cancer therapeutics targeting lipid metabolism.

Agent	Metabolic target	Indications	Concentration/dose of agent	Study model	Outcomes	References
SB‐2049990	ACLY inhibitor	Lung carcinoma	25 mg/kg	In vivo, *xenograft*	Inhibited lipid synthesis, proliferation, xenograft tumour formation o lung cancer	[23, 191]
ND‐654	ACC inhibitor	NSCLC, HCC	10 mg/kg	In vivo	Inhibited lipogenesis & HCC development.	[192]
ND‐646	ACC inhibitor	NSCLC	1 μM	In vitro	Induces cancer cell death. Inhibits tumour progression.	[193]
TOFA	ACC inhibitor	Head and neck SCC ovarian carcinoma, colon carcinoma, RCC	0–50 μm; 50 mg/kg	In vitro and in vivo	Cell cycle arrests & apoptosis induction. Significantly inhibits tumour growth rate in ovarian tumour mouse xenografts.	[194]
Metformin	ACC inhibitor	Lung carcinoma, prostate carcinoma, ovarian carcinoma	0–50 mM; 3 mg in 100 μL PBS/day	Both in vivo and in vitro	Reduces cell viability and proliferation. Inhibits ERK1/2 and mTOR phosphorylation/activation, and stimulates AMPK activity. Inhibits tumor growth	[195]
BZ36	Inhibitor palmitate	Ovarian carcinoma	1–5 μM; 1 mg/kg	Both in vivo and in vitro	Enhances ferroptosis Induces antitumor effect	[103]
Anti‐CD36 antibody	Fatty acid uptake inhibitor	Stomach carcinoma	20 μg/100 μL	In vivo	Poor metastasis	[196]
Statins	HMG‐CoA reductase inhibitor	Breast carcinoma	Registry dose	Population based	Induces cancer cell death.	[197]
C75	FASN inhibitor	Melanoma, breast, lung carcinomas, glioblastoma, RCC, mesothelioma, gliomas	30 mg/kg	In vivo	Greatly delayed tumor progression	[198, 199]
Cerulenin	FASN inhibitor	Ovarian carcinoma, breast carcinoma	0–40 μM	In vitro	Induced apoptosis	[199, 200]
Orlistat	FASN inhibitor	Prostate carcinoma, melanoma, glioma endometrial carcinoma, melanoma, tongue SCC	25 μM; 240 mg/kg	Both in vivo and in vitro	Stops tumor cell proliferation and Induces a poptosis	[199, 201]
TVB2640	FASN inhibitor	NSCLC, colorectal carcinoma, breast carcinoma, astrocytoma	100 mg/m^2^	Clinical trials	Inhibits fatty acid de novo synthesis	[23, 202]
Triclosan	FASN inhibitor	Prostate carcinoma, breast carcinoma	N/A	In vitro	Changes in morphology, cell cycle, lipid content, and enzyme expression	[199, 203]
Amento‐flavone	FASN inhibitor	Breast carcinoma	50, 75 and 100 μM	In vitro	Induced apoptosis	[204]
EGCG	FASN inhibitor	Lung carcinoma, breast carcinoma	150 μM and 30 mg/kg	Both in vivo and in vitro	Stimulated apoptosis. Reduced HER2‐positive cells' growth	[205]
TVB‐3166	FASN inhibitor	Ovarian carcinoma	0–100 μM	In vitro	Inhibit cell proliferation and induce apoptosis	[206]
BZ36	SCD inhibitor	Prostate carcinoma	80 mg/kg	In vivo	Blocked oncogenesis and cancer progression.	[207]
A939572	SCD inhibitor	Renal carcinoma	Not mention and 10 mg/kg	Both in vivo and in vitro	Reduced cancer cell proliferation	[208]
CAY‐10566	SCD inhibitor	HCC, breast carcinoma, head and neck SCC, ovarian carcinoma	2.5 mg/kg	In vivo	Inhibit lipogenesis. Ras‐driven cells are resistant to SCD1 inhibition in culture and allografts.	[209]
CVT‐11127	SCD inhibitor	HCC, lung carcinoma	1.00 μM	In vitro	Active SCD1 controls glucose‐mediated lipogenesis. Impaired SCD1 activity downregulates Impaired SCD1 activity downregulates Impaired SCD1 activity downregulates SFA synthesis. AMPK‐mediated inactivation prevents harmful SFA accumulation effects	[210]
MF‐438	SCD inhibitor	Lung carcinoma	5 mg/kg	In vivo	Potent SCD inhibitor. Exhibits good pharmacokinetics and metabolic stability	[211]
T‐3764518	SCD inhibitor	CRC, mesothelioma	(1,10,100) nM; (o.3, 3.0, 30) mg/kg	In vitro and in vivo	Reduce cell proliferation	[212]
Etomoxir	CPT1 inhibitor	Prostate carcinoma, breast carcinoma, glioblastoma	50 μM	In vitro	Induces severe oxidative stress in T cells Showed off‐target effects	[213]
Ranolazine	CPT1 inhibitor	Prostate carcinoma	50 μM, 35 mg/kg	In vitro and in vivo	Lacked fatty acid β‐oxidation‐interfering activity	[214]
Perhexiline	CPT1 inhibitor	Breast cancer, prostate cancer, lymphocytic leukemia	5–25 μM; 80 mg/kg	Both in vivo and in vitro	Reduced cell viability, ATP content, and LDH release. Induced massive apoptosis	[215, 216]
Triacscin C	ACSL inhibitor	Lung carcinoma, CRC, brain cancer	5.0 μM	In vitro	Induced apoptosis through a mitochondrial pathway	[217]

Abbreviations: ACC, acetyl‐CoA carboxylase; ACLY, ATP‐citrate lyase; ACSL, long‐chain fatty acyl‐CoA synthetase; AMPK, AMP‐activated protein kinase; CD36, cluster of differentiation 36; CRC, colorectal cancer; EGCG, epigallocatechin‐3‐gallate; ERK, extracellular signal‐regulated kinase; FASN, fatty acid synthase; HCC, hepatocellular carcinoma; HMG‐CoA, β‐hydroxy β‐methylglutaryl‐CoA; LDH, lactate dehydrogenase; NSCLC, nonsmall‐cell lung cancer; PARP, poly[ADP‐ribose] polymerase; RCC, renal cell carcinoma; SCC, squamous cell carcinoma; SCD, stearoyl‐CoA desaturase; SFA, saturated fatty acid; TOFA, 5‐(tetradecyloxy)‐2‐furoic acid.

#### 7.3.1 Therapeutic Targeting De Novo Lipogenesis

The initial stage in fatty acid production is acetyl‐CoA generation, followed by malonyl‐CoA synthesis by ACC, which is essential for carcinogenesis. Mammals have two subtypes of ACC (ACC1 and ACC2), which are enzymes that limit the rate at which fatty acids are synthesized. ACC1 contributes to fat formation and is required for lipid generation, whereas ACC2 is primarily engaged in lipid oxidation. Malonyl‐CoA concentrations only decline when both ACC1 and ACC2 are inactive because when one stops working, the other can compensate [218]. Myc‐induced kidney tumors were treated with 5‐(tetradecyloxy)‐2‐furoic acid (TOFA), an ACC1 inhibitor, which induced tumor regression [22]. ND646, another ACC inhibitor, induced a reduction of both tumor fatty acid production and tumor development of A549 xenograft and KRAS‐driven lung carcinomas [219, 220]. In clinical models, it was noted that ACC mediates the metabolism of NSCLC and that ND‐646 inhibits the growth of NSCLC [193]. Mimicking ACC phosphorylation or the ACC inhibitor, ND‐654, can also block the formation of new fatty acids in the liver, reducing the rate of growth and development of liver cancer cells [23]. Research has indicated that ACC suppression will also have an impact on the viability of pancreatic cancer cells [221].

C75, a cerulenin‐derived natural product, acts as a FASN antagonist obtained from *Cephalosporium caerulens* [222]. It was noted that C75 exhibited in vivo antitumor activities in preclinical studies [198, 222, 223]. However, its unwanted side effects limit its potential for clinical use [198, 223]. Another FASN inhibitor, TVB‐3166, has shown effectiveness in NSCLC xenograft models in combination with taxane [224]. Additionally, TVB‐2640, another FASN inhibitor, is currently being evaluated in clinical trials for several solid cancers, including NSCLC [202], HER2+ advanced‐stage breast carcinoma [225], and high‐grade astrocytoma [226]. The field is eagerly anticipating the findings of these investigations, decades after FASN was discovered as a promising target for therapy.

ACLY is a crucial enzyme that catalyzes the transformation of acetyl‐CoA into fatty acids. It links the energy created throughout the catabolic and biosynthetic processes to ensure the sustainability of cancer cells' activities. Since ACLY is typically overexpressed in a range of cancers, one cancer treatment option is to alter the enzyme's citric acid binding site through indirect transcyclic citric acid‐binding. SB‐204990, an ACLY inhibitor, has a potent inhibitory effect on lung carcinoma, prostate carcinoma, and other mouse tumors [23].

SCD1 is a major anticancer target because it regulates cancer cell proliferation, migration, and invasion. In ovarian carcinoma, SCD1 is highly expressed in a variety of ovarian carcinomas and protects them from death [11]. In an ovarian tumor model, BZ36 inhibits SCD1 synthesis, sensitizing ovarian cancer cells to ferroptosis‐inducing drugs and causes tumor cell death [103]. Presently, several drugs targeting SCD1 in tumor treatment, such as CAY‐10566 and MF‐438, have only been tested in preclinical trials, primarily owing to weight loss and severe adverse effects on the skin and eyes [227]. Preventing these harmful side effects is critical in the growth of the next generation of SCD1 blockers [228].

#### 7.3.2 Therapeutic Targeting Fatty Acid Update

Exogenous fatty acid intake can cause overexpression of CD36 in malignancies, including breast carcinoma, stomach carcinoma, and ovarian carcinoma. CD36 deficiency in mouse prostate tissue can inhibit cancer uptake of fatty acid organisms and decrease tumorigenesis [23]. CD36 expression increased dramatically in breast cancer patients after receiving anti‐HER2 medication. Last, an anti‐CD36 antibody has potent antitumor action in human melanoma cells [229].

HMG‐CoA reductase is an important rate‐limiting enzyme in cholesterol production. It transforms HMG‐CoA into mevalonate. Overexpression of HMG‐CoA reductase promotes tumor cell proliferation and metastasis; hence, blocking this enzyme is used as an approach for developing cancer drugs [23]. Rewiring the metabolic process of cholesterol inside and outside the cell in the TME can increase the growth of colon, breast, and prostate carcinomas [23]. Statins block HMG‐CoA reductase, and in statin‐treated breast carcinoma, the absence or low expression of HMG‐CoA reductase implies favorable clinical outcomes for patients with the carcinoma. Pitavastatin, a novel cholesterol medication, influences cholesterol metabolism and induces apoptosis in colon cancer stem cells [230].

Fatty acids provide a variety of activities in cells, one of which is to supply energy via fatty acid β‐oxidation. Overexpression of enzymes related to fatty acid β‐oxidation has been linked to several cancers and has been shown to play a key role in tumor formation [199]. CPT1, a rate‐limiting enzyme in fatty acid β‐oxidation, is essential for cancer cell growth [199]. CPT1 blockers, for instance, etomoxir and ranolazine, exhibit anticancer activity in prostate carcinomas [142]. A similar outcome has been seen in breast carcinoma [231] and glioblastoma [232]. In combination with therapy, etomoxir can increase the effectiveness of chemotherapy for patients with lymphoma [232].

### 7.4 Cancer Therapeutics Targeting Nucleotide Metabolism

Nucleotide metabolism is significant in cancer growth and progression. Targeting nucleotide metabolism has been the focus of much research and development in cancer treatment [233]. Over 20 nucleotides and nucleotide analogs have been licensed for use in cancer chemotherapies, accounting for ~20% of all cancer drugs (Table 4). Based on their structures and actions, therapeutic medicines targeting nucleotide metabolism are categorized into three classes: purine analogs, pyrimidine analogs, and metabolic enzymatic blockers [233]. Examples of purine analog antimetabolites are thiopurines, deoxypurines, arabinose purine analogs, and purine nucleosides with base modifications [282]. In the early 1950s, Elion's group established that 6‐mercaptopurine and thioguanine, two of the first thiopurine analogs identified, could inhibit *Lactobacillus casei* development [277]. In 1953, 6‐mercaptopurine was licensed by the FDA for the treatment of juvenile leukemia after clinical trials showed its efficacy in suppressing nucleoside phosphorylation and hydrolysis [241, 283]. Methotrexate inhibits dihydrofolate reductase and has played an important role in the progress of cancer therapies. Cladribine, an authorized deoxyadenosine analog, was utilized as the first‐line treatment for hairy cell leukemia [256]. In addition, Clofarabine, a second‐generation deoxyadenosine, was prescribed for relapsed or refractory juvenile acute lymphoblastic leukemia [269, 270, 284]. Nelarabine and fludarabine are two arabinose purine analogs. They were authorized for the management of chronic lymphocytic leukemia, relapsed T‐cell lymphoblastic lymphoma, and relapsed T‐cell acute lymphoblastic leukemia [120].

**TABLE 4:** Cancer therapeutics targeting nucleotide metabolism.

Agent	Metabolic target	Indications (all FDA‐approved)	Formulation(s)	Outcomes	References
6‐Mercaptopurine (6‐MP)	Inhibitor of hypoxanthine–guanine phosphoribosyl‐transferase, amidophosphoribosyl‐transferase	ALL	Oral: 50 mg tablet Oral: 20 mg per mL	Interferes with nucleic acid synthesis	[233, 234, 235, 236]
Methotrexate	Inhibitor of dihydrofolate reductase, thymidylate synthase, amido phosphoribosyl‐transferase & aminoimidazole carboxamide ribonucleotide transformylase	ALL, gestational choriocarcinoma, invasive mole hydatidiform mole, breast, SCC, lung carcinomas, and advanced non‐Hodgkin's lymphomas	Parenteral: 50 mg in vial Oral: 2.5 mg Parenteral‐ IV: 50 mg in vial; 50 mg per 2 mL; 1000 mg per 10 mL concentrated injection	Interferes with nucleotide synthesis	[237, 238]
5‐Fluorouracil	Inhibitor of thymidylate synthase	Carcinomas of colon, cervix, esophagus, rectum, breast, RCC, stomach, head and neck, pancreas, and biliary tract	Parenteral–IV: 50 mg per mL in 5 mL ampoule/vial	Interferes with DNA synthesis	[233, 239, 240]
Thioguanine	DNA intercalation as a false purine base	Acute non‐lymphocytic leukemia	Oral—Solid: 40 mg	Prevents normal development and division of cells	[233, 241, 242, 243]
Cytarabine	Inhibitor of DNA polymerase	Acute non‐lymphocytic leukemia	Parenteral: 100 mg in a vial powder for injection	Damages DNA, inhibits DNA repair and cell division.	[244, 245]
Floxuridine	Inhibitor of thymidylate synthase Pyrimidine analog	Gastrointestinal tract adenocarcinoma, liver carcinoma	Powder, 500 mg in 5‐mL containers	Disrupts S‐phase of cell division. Being a pyrimidine analog, prevent normal pyrimidines from being incorporated into DNA, preventing DNA synthesis.	[233, 246, 247, 248]
Cisplatin	DNA cross‐linking/alkylation	Testicular tumor, ovarian carcinoma, and bladder carcinoma	Parenteral: 10–50 mg in a vial powder for injection	Inhibits tumor growth by cross‐linking guanine bases in DNA, preventing strand separation and replication, and thus cell division. Adds alkyl groups to nucleotides, disrupting proper base pairing, leading to DNA miscoding.	[249, 250]
Carboplatin	DNA cross‐linking/alkylation	Advanced ovarian carcinoma	Parenteral: 50 mg/5 mL	Same as cisplatin	[251, 252]
Fludarabine	Inhibitor of ribonucleoside‐diphosphate reductase large subunit and DNA polymerase alpha catalytic subunit	CLL	Parenteral—IV: 50 mg in a vial; Oral—Solid: 10 mg tablet (fludarabine phosphate)	Inhibits DNA synthesis	[233, 253, 254, 255]
Cladribine	Inhibitor of ribonucleoside‐diphosphate reductase and DNA polymerase	Hairy cell leukaemia, CLL, non‐Hodgkin's lymphoma	Parenteral—IV: 1 mg/10 mL and 2 mg/5 mL vial	Leads to DNA‐strand breaks, causing apoptosis, or autophagy in dividing and resting lymphocytes.	[233, 256, 257]
Pentostatin	Inhibitor of adenosine deaminase	Hairy cell leukaemia	Parenteral: for injection, 10 mg pentostatin (with mannitol 50 mg)	Same as thioguanine	[233, 258, 259]
Hydroxyurea	Inhibitor of ribonucleoside reductase	CML, AML, head and neck SCC	Oral capsules –200 mg, 250 mg, 300 mg, 400 mg, 500 mg, 1 g	Inhibits DNA synthesis	[260, 261]
Gemcitabine	Inhibitor of UMP‐CMP kinase, ribonucleoside‐diphosphate reductase, and thymidylate synthase	Ovarian, breast, pancreatic carcinomas, and NSCLC	Parenteral–IV: 200–1000 mg in a vial powder for injection	Blocks progression of cells through G1/S‐phase boundary, promoting apoptosis of malignant cells undergoing DNA synthesis.	[233, 262, 263, 264]
Dacarbazine	DNA cross‐linking/alkylation	Melanoma, Hodgkin lymphoma	Parenteral—IV: 100–200 mg in vial powder for injection	Adds alkyl groups to nucleotides. Being purine analog, inhibits DNA synthesis.	[265, 266]
Capecitabine	Inhibitor of thymidylate synthase	Breast carcinoma and colon carcinoma	Oral—Solid—tablet: 150 mg; 500 mg	Prodrug of 5‐fluorouracil. Impairs DNA synthesis and repair, causing apoptosis.	[267, 268]
Clofarabine	Inhibitor of ribonucleoside‐diphosphate reductase, DNA polymerase	ALL	20 mg/20 mL	Inhibits DNA and RNA synthesis.	[269, 270, 271]
Azacytidine	Inhibit DNA methylation	Chronic myelomono‐cytic leukaemia	75 mg/m^2^ subcutaneous	Inhibits DNA synthesis. Induces cytotoxicity by incorporating itself into RNA and DNA.	[233, 272, 273, 274]
Nelarabine	Inhibitor of DNA polymerase, DNA ligase 1, DNA primase, and ribonucleoside diphosphate reductase	Acute T‐cell lympho‐blastic leukaemia, T‐cell lympho‐blastic lymphoma	5 mg/mL	DNA fragmentation and cell apoptosis	[233, 275, 276]
Decitabine	Inhibit DNA (cytosine‐5)‐methyl‐transferase 1, 3A and 3B	Myelo‐dysplastic syndromes	50 mg injection or powder	Incorporates into DNA and exerts numerous effects on gene expression	[233, 277]
Oxaliplatin	DNA cross‐linking/alkylation	CRC	Parenteral—IV: 50–200 mg in vial powder for injection	Forms DNA cross‐linking, inhibiting DNA replication and transcription	[278, 279]
Pemetrexed	Inhibitor of thymidylate synthase and dihydrofolate reductase	Meso‐thelioma, NSCLC	Powder lyophilized: 500 mg; 500 mg mannitol	Inhibits the nucleotides biosynthesis	[280, 281]

Abbreviations: 6‐MP, 6‐mercaptopurine; ALL, acute lymphoblastic leukemia; AML, acute myeloid leukemia; CLL, chronic lymphocytic leukemia; CML, chronic myeloid leukemia; CRC, colorectal carcinoma; DNA, deoxyribonucleic acid; NSCLC, non‐small cell lung cancer; RCC, renal cell carcinoma; SCC, squamous cell carcinoma.

Exogenous uracil is used for nucleic acid production throughout the in vivo phase of hepatic carcinogenesis, according to observations made by Rutman et al. in 1954 [285]. Heidelberger et al. [286] produced fluorouracil based on their finding and previous research on TYMS. Chemotherapeutic drugs, such as gemcitabine and 5‐fluorouracil, are essential because they impede nucleotide metabolism [227, 239]. Subsequently, the FDA approved 5‐fluorouracil in 1960, and it is now utilized to treat a variety of malignancies, including gastrointestinal carcinomas, breast carcinoma, and renal cell carcinoma [239, 287, 288, 289, 290]. Hertel et al. [291] developed gemcitabine, a pyrimidine antimetabolite, in 1990 and demonstrated its potent anticancer efficacy in experimental tumor models. Gemcitabine was initially investigated in hematological cancers and discovered to have excellent anticancer properties, not only in hematological cancers but also in other solid tumors [282]. Floxuridine is transformed into floxuridine‐5′‐monophosphate II via thymidine kinase; hence, floxuridine inhibits TYMS, and the FDA approved it for metastatic colorectal malignancies in 1970 [292]. Decitabine and azacytidine are azanucleosides that block DNA methylation for anticancer effects [233]. Gemcitabine, a ribosugar‐modified cytidine analog, inhibits DNA manufacturing by inducing cell cycle arrest by “masked chain termination” [293]. Cytarabine was discovered to exhibit anticancer properties in vivo in the 1960s, investigated quickly in animal models and clinical studies, and was subsequently approved by the FDA [294]. Pentostatin, a deoxyadenosine drug that targets adenosine deaminase, has received FDA approval for hairy cell leukemia [258]. Based on the review of therapeutic drugs targeting nucleotide metabolism, this study emphasizes the importance and dependability of pursuing nucleotide metabolism as chemotherapy in malignancies. These findings have not only considerably improved our understanding of the regulation mechanism of nucleotide metabolism in malignancies but have also given insights into the clinical trials of new specialized therapeutic medicines. Although chemotherapy remains the foundation of all cancer treatment regimens in the adjuvant setting, its efficacy is limited by drug resistance and associated significant adverse effects.

**FIGURE 4:**
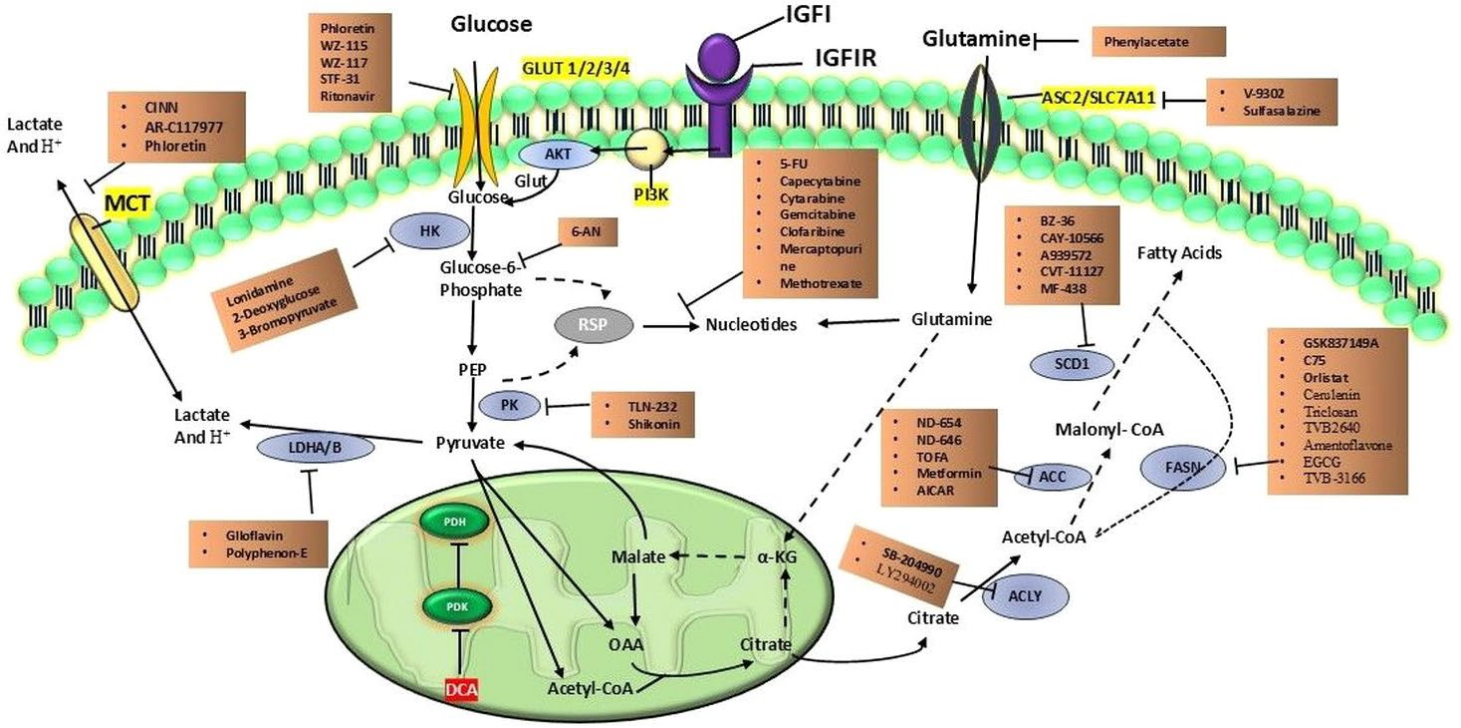
Potential therapeutics targeting cancer metabolism. This figure demonstrates the metabolic pathways and enzymes against which drugs have already been tested in clinical trials. Several pathways of malignant cell bioenergetic and anabolic metabolism provide potential cancer therapy targets. In general, drugs that disrupt these pathways are likely to cause deficits in energy and materials required for cell proliferation and survival, laying the groundwork for their application as anticancer treatments.

In summary, as cancer cells exhibit metabolic plasticity, several therapeutic strategies are being developed to target the metabolic adaptation of cancer cells (Figure 4).

## 8 Current Limitations and Future Perspectives

Current limitations in targeting metabolic reprogramming in cancer cells primarily stem from the complexity and heterogeneity of tumor metabolism, which can vary significantly between different cancer types. Although this review addresses the various metabolic reprogramming in cancer cells and their implications for therapeutic development, it does not extensively address the heterogeneity of cancer types, which may lead to varying therapeutic responses. This variability poses challenges in developing universally effective therapies that can consistently target altered metabolic pathways. Moreover, while targeting metabolic processes shows promise, such approaches can inadvertently lead to drug resistance as cancer cells may adapt by exploiting alternate pathways for survival. Additionally, while this review discusses the altered mechanisms of the metabolic pathways, the interplay between metabolic reprogramming in the cell and the tumor microenvironment is not sufficiently explored. Last, this review could also benefit from more discussion on the clinical challenges associated with the therapeutics mentioned, especially concerning potential side effects.

Future perspectives involve a more nuanced understanding of metabolic networks within individual tumors, leveraging advanced technologies to personalize treatment approaches. As research into cancer cell metabolism deepens, this knowledge can be used to design targeted interventions that disrupt more specific metabolic processes involving glucose, amino acid, lipid, and nucleotide metabolism. Moreover, by continuing to discover combination therapies, especially with emerging immunotherapy treatments, more therapy strategies can be utilized to combat drug resistance. The potential to personalize cancer therapies based on an individual's tumor metabolic profile could lead to more effective and less toxic treatment options. Furthermore, ongoing research into the communication between metabolic pathways and the tumor microenvironment may reveal novel targets to disrupt tumor growth and survival. Ultimately, these advancements may not only improve patient outcomes but also pave the way for a new era of precision medicine in oncology.

## 9 Conclusion

There are several modifications made in the metabolic processes of cancer cells that allow them to sustain proliferation. The overarching metabolic processes discussed in this review concern glucose, amino acid, lipid and nucleotide metabolism. Examples of the alterations seen in cancer cells regarding these processes involve changes to the transport of molecules, enzyme activity and receptor expression. Cancer therapeutics have also been developed and are currently being researched to target altered metabolic processes. However, these alterations to the metabolic processes pose a challenge to cancer drug resistance; thus, targeting these processes in combination with the usual drug therapy may aid in overcoming therapy resistance.

## Author Contributions

**Jilsy M. J. Punnasseril:** data analysis and writing the draft. **Abdul Auwal:** data curation, writing – original draft preparation. **Vinod Gopalan:** supervision. **Alfred King‐Yin Lam:** editing. **Farhadul Islam:** writing – reviewing and editing, supervision.

## Conflicts of Interest

The authors declare no conflicts of interest.

## Data Availability

Data sharing not applicable to this article as no datasets were generated or analysed during the current study.
